# Pectus excavatum and chest pain: a case report

**DOI:** 10.1186/1757-1626-1-351

**Published:** 2008-11-25

**Authors:** Regina Y Kim, Craig J Baker

**Affiliations:** 1 University of Southern California Keck School of Medicine, Division of Plastic and Reconstructive Surgery, 1510 San Pablo Street, suite 415. Los Angeles, CA 90033, USA; 2 University of Southern California Keck School of Medicine, Cardiovascular and Thoracic Institute, 1520 San Pablo Street, suite 4300. Los Angeles, CA 90033, USA

## Abstract

**Background:**

Simultaneous repair of pectus deformities and open heart surgery has been reported.

**Case Presentation:**

We describe a case of a 23 year old male with a severe pectus deformity complaining of chest pain. Subsequent work up revealed a 10 cm. ascending aortic aneurysm and severe aortic insufficiency. We opted to repair the aneurysm and close the sternotomy, leaving the deformity intact.

**Conclusion:**

In a more elective setting, simultaneous pectus repair combined with a cardiac procedure may be considered.

## Background

Simultaneous repair of pectus deformities and open heart surgery has been reported. Controversy exists regarding the repair of the pectus deformity at the time of the cardiac procedure.

## Case presentation

**Figure 1:**
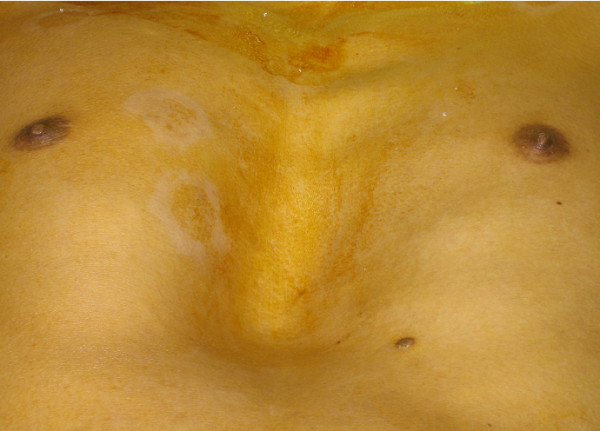
**Pectus excavatum deformity.** Physical examination of the patient demonstrating a pectus excavatum deformity.

**Figure 2:**
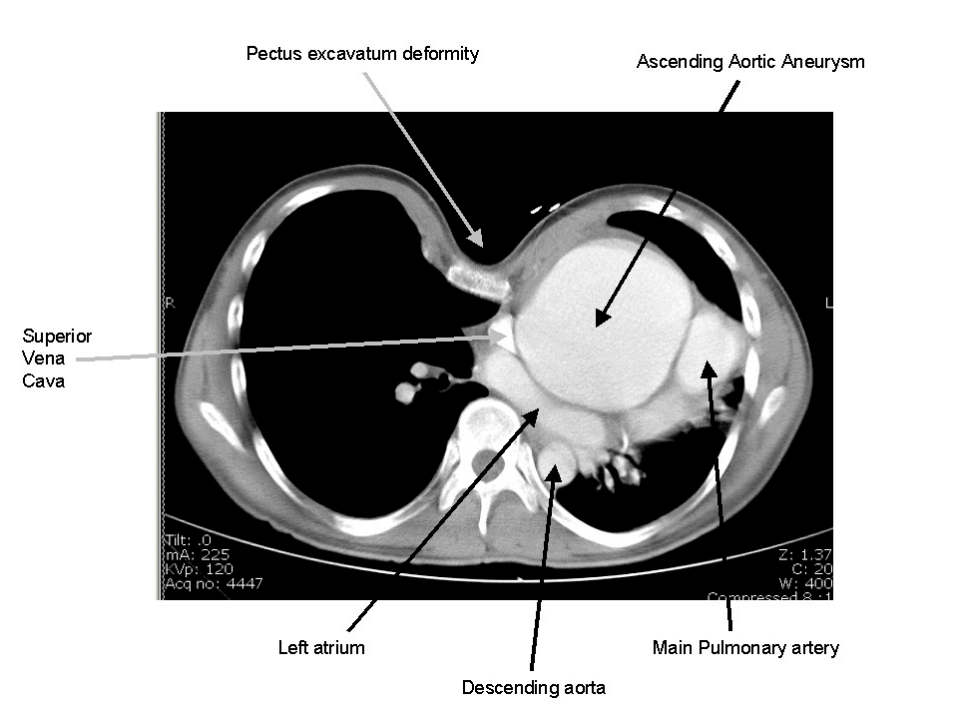
**CT scan.** CT scan demonstrating a 10 cm ascending aortic aneurysm. The surrounding vascular structures are labelled. Note the leftward displacement of the mediastinal structures secondary to the pectus deformity.

**Figure 3:**
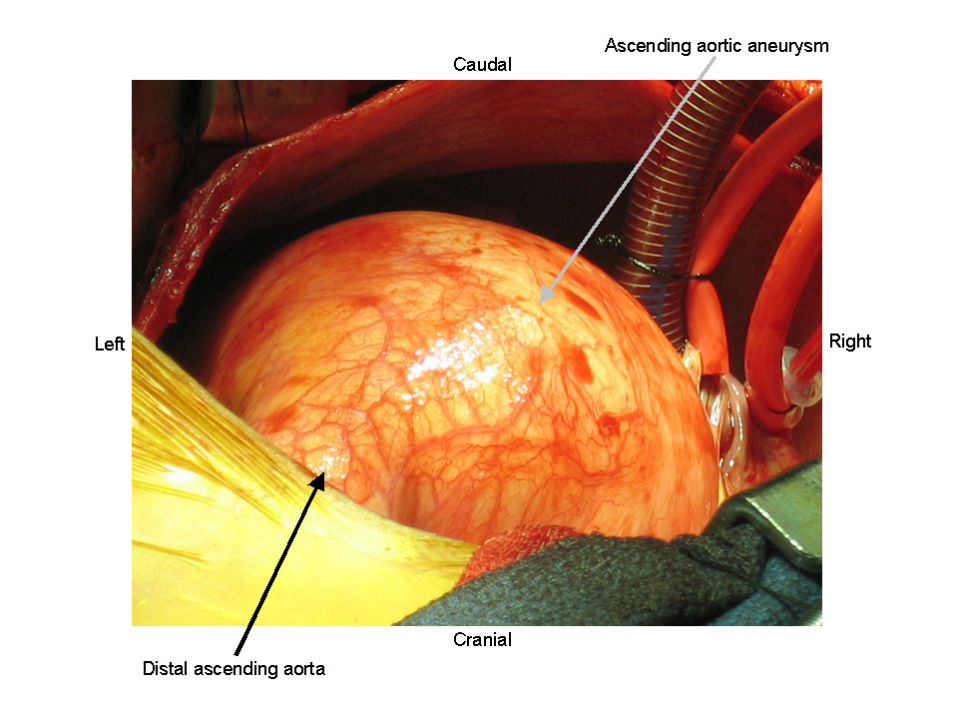
**Operative photograph.** Intra-operative photograph demonstrating the large ascending aortic aneurysm prior to repair. The distal ascending aorta is noted to be normal.

A 23 year-old Hispanic male, with no prior medical history, presented to the emergency department with a two-week history of increasing chest pain. His physical examination revealed a significant pectus excavatum deformity (Figure 1) and a diastolic murmur. Other physical findings were suggestive of Marfan's disease. He denied alcohol or tobacco use. An abnormal chest x-ray prompted further work up with a CT scan that demonstrated leftward displacement of the heart and great vessels, as well as 10 cm ascending aortic aneurysm (Figure 2). An echocardiogram confirmed severe aortic valve insufficiency. The patient underwent aortic root replacement and aneurysm repair with a valved conduit. An operative photograph exhibits the large ascending aneurysm and a normal appearing distal ascending aorta (Figure 3). The patient made an uneventful postoperative recovery.

## Discussion

There are numerous case reports documenting simultaneous repair of cardiovascular disorders and correction of pectus deformities [1-6]. In the above case, we opted to close the sternotomy and leave the deformity intact. The patient stated he had no previous problems related to his deformity and no desire for pectus repair. This decision was facilitated by the potential risk of wound complications in the setting of a newly placed synthetic graft used for the aortic root replacement.

## Conclusion

In a more elective setting, simultaneous pectus repair combined with a cardiac procedure may be considered.

## Consent

"Written informed consent was obtained from the patient for publication of this case report and accompanying images. A copy of the written consent is available for review by the Editor-in-Chief of this journal."

## Competing interests

The authors declare that they have no competing interests.

## Authors' contributions

RK and CB equally contributed to the preparation of this manuscript. RK and CB both approved the final manuscript.
